# Complete genome sequence of the hyperthermophilic chemolithoautotroph *Pyrolobus fumarii* type strain (1A^T^)

**DOI:** 10.4056/sigs.2014648

**Published:** 2011-07-01

**Authors:** Iain Anderson, Markus Göker, Matt Nolan, Susan Lucas, Nancy Hammon, Shweta Deshpande, Jan-Fang Cheng, Roxanne Tapia, Cliff Han, Lynne Goodwin, Sam Pitluck, Marcel Huntemann, Konstantinos Liolios, Natalia Ivanova, Ioanna Pagani, Konstantinos Mavromatis, Galina Ovchinikova, Amrita Pati, Amy Chen, Krishna Palaniappan, Miriam Land, Loren Hauser, Evelyne-Marie Brambilla, Harald Huber, Montri Yasawong, Manfred Rohde, Stefan Spring, Birte Abt, Johannes Sikorski, Reinhard Wirth, John C. Detter, Tanja Woyke, James Bristow, Jonathan A. Eisen, Victor Markowitz, Philip Hugenholtz, Nikos C. Kyrpides, Hans-Peter Klenk, Alla Lapidus

**Affiliations:** 1 DOE Joint Genome Institute, Walnut Creek, California, USA; 2 DSMZ - German Collection of Microorganisms and Cell Cultures GmbH, Braunschweig, Germany; 3 Los Alamos National Laboratory, Bioscience Division, Los Alamos, New Mexico, USA; 4 Biological Data Management and Technology Center, Lawrence Berkeley National Laboratory, Berkeley, California, USA; 5 Oak Ridge National Laboratory, Oak Ridge, Tennessee, USA; 6 University of Regensburg, Microbiology – Archaeenzentrum, Regensburg, Germany; 7 HZI – Helmholtz Centre for Infection Research, Braunschweig, Germany; 8 University of California Davis Genome Center, Davis, California, USA; 9 Australian Centre for Ecogenomics, School of Chemistry and Molecular Biosciences, The University of Queensland, Brisbane, Australia

**Keywords:** hyperthermophile, chemolithoautotroph, facultative microaerophilic, non-motile, hydrothermal solfataric vents, black smoker, *Pyrodictiaceae*, GEBA

## Abstract

*Pyrolobus fumarii* Blöchl *et al*. 1997 is the type species of the genus *Pyrolobus*, which belongs to the crenarchaeal family *Pyrodictiaceae*. The species is a facultatively microaerophilic non-motile crenarchaeon. It is of interest because of its isolated phylogenetic location in the tree of life and because it is a hyperthermophilic chemolithoautotroph known as the primary producer of organic matter at deep-sea hydrothermal vents. *P. fumarii* exhibits currently the highest optimal growth temperature of all life forms on earth (106°C). This is the first completed genome sequence of a member of the genus *Pyrolobus* to be published and only the second genome sequence from a member of the family *Pyrodictiaceae*. Although Diversa Corporation announced the completion of sequencing of the *P. fumarii* genome on September 25, 2001, this sequence was never released to the public. The 1,843,267 bp long genome with its 1,986 protein-coding and 52 RNA genes is a part of the *** G****enomic* *** E****ncyclopedia of* *** B****acteria and* *** A****rchaea * project.

## Introduction

Strain 1A^T^ (= DSM 11204) is the type strain of the species *Pyrolobus fumarii*, which is the type and only species of its genus *Pyrolobus* [1]. The generic name derives from the Greek word *pyr* meaning *fire* and the Greek word *lobos* meaning *lobe*, referring to fire lobe. The species epithet is derived from the Latin word *fumarii* meaning *of the chimney*, referring to its black smoker biotope [1]. Strain 1A^T^ was isolated from a black smoker wall, TAG site, Mid Atlantic Ridge [2], effectively published in 1997 [1] and validly published in 1999 [3]. It is thus far the most heat-tolerant, and also most heat-requiring of all validly named prokaryotic species. *P. fumarii* appears to be the primary producer of organic material in such deep-sea hydrothermal vent habitats [1]. At the time of its discovery, *P. fumarii* extended the upper temperature limit for life to 113°C [1]. A more recent report on a not yet validly named and incompletely characterized iron-reducing archaeon, known only as ‘Strain 121’ appears to extend the upper growth temperature to 121°C, which is well within standard autoclaving temperatures [4]. Here we present a summary classification and a set of features for *P. fumarii* strain 1A^T^, together with the description of the complete genomic sequencing and annotation.

## Classification and features

The single genomic 16S rRNA sequence of strain 1A^T^ was compared using NCBI BLAST [5] under default settings (e.g., considering only the high-scoring segment pairs (HSPs) from the best 250 hits) with the most recent release of the Greengenes database [6] and the relative frequencies of taxa and keywords (reduced to their stem [7]) were determined, weighted by BLAST scores. The most frequently occurring genera were *Aeropyrum* (18.1%), *Desulfurococcus* (11.1%), *Ignicoccus* (9.8%), *Vulcanisaeta* (7.8%) and *Staphylothermus* (7.0%) (68 hits in total). Regarding the single hit to sequences from members of the species, the average identity within HSPs was 99.0%, whereas the average coverage by HSPs was 46.1%. Among all other species, the one yielding the highest score was *Hyperthermus butylicus* (NC_008818), which corresponded to an identity of 99.2% and an HSP coverage of 46.1%. (Note that the Greengenes database uses the INSDC (= EMBL/NCBI/DDBJ) annotation, which is not an authoritative source for nomenclature or classification.) The highest-scoring environmental sequence was AB293243 ('Microbial structures around area Southern Mariana Trough hydrothermal sulfide structure clone Pcsc3A31'), which showed an identity of 96.9% and an HSP coverage of 44.7%. The most frequently occurring keywords within the labels of environmental samples which yielded hits were 'spring' (12.0%), 'hot' (7.5%), 'microbi' (7.0%), 'nation, park, yellowston' (6.2%) and 'geochem' (3.7%) (181 hits in total). Environmental samples which yielded hits of a higher score than the highest scoring species were not found. These keywords reflect some of the ecological features and properties reported for strain 1A^T^ in the original description [1].

**Figure 1:**
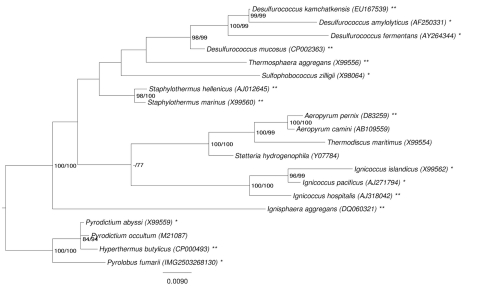
Phylogenetic tree highlighting the position of *P. fumarii* relative to the type strains of the other species within the order *Desulfurococcales*. The tree was inferred from 1,333 aligned characters [8,9] of the 16S rRNA gene sequence under the maximum likelihood (ML) criterion [10]. Rooting was done initially using the midpoint method [11] and then checked for its agreement with the current classification (Table 1). The branches are scaled in terms of the expected number of substitutions per site. Numbers adjacent to the branches are support values from 1,000 ML bootstrap replicates [12] (left) and from 1,000 maximum parsimony bootstrap replicates [13] (right) if larger than 60%. Lineages with type strain genome sequencing projects registered in GOLD [14] are labeled with one asterisk, those also listed as 'Complete and Published' with two asterisks (see [15-21], and CP002051 for *Staphylothermus hellenicus*).

Figure 1 shows the phylogenetic neighborhood of *P. fumarii* in a 16S rRNA based tree. The sequence of the single 16S rRNA gene copy in the genome does not differ from the previously published 16S rRNA sequence (X99555), which contains eleven ambiguous base calls.

**Figure 2:**
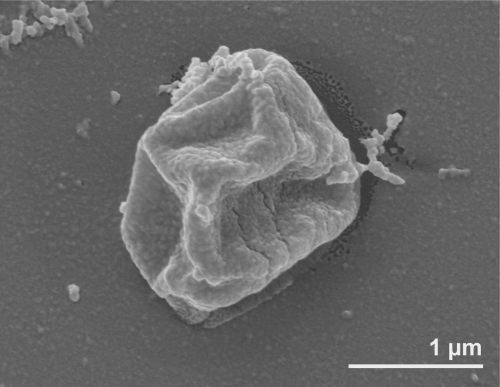
Scanning electron micrograph of *P. fumarii* 1A^T^

Cells of strain 1A^T^ are regularly to irregularly lobed cocci with a diameter of approximately 0.7-2.5 µm (Figure 2) [1]. The strain is non-motile, non-spore-forming and facultatively microaerophilic (Table 1). Strain 1A^T^ has a temperature range for growth between 90°C and 113°C (optimum 106°C) and is unable to propagate at a temperature of 90°C or below [1,32]. Exponentially growing cultures of *P. fumarii* survive even autoclaving at 121°C for one hour [1]. At the optimum growth temperature, doubling time of *P. fumarii* is 60 minutes [1]. The pH range for growth is 4.0-6.5, with an optimum pH of 5.5 [1]. The strain forms white colonies (1 mm in diameter) on Gelrite-containing media [1]. Like in *Hyperthermus*, no cell-to-cell network is formed and the S-layer exhibits a central depression, most likely a pore [1,32]. Such networks of extracellular tubules appear to be characteristic for members of the genus *Pyrodictium*. *P. fumarii* strain 1A^T^ is able to grow on medium that contains 1%-4% NaCl, with an optimum salinity at 1.7% [1]. The organism uses CO_2_ as the single carbon source and H_2_ as the obligate electron donor [1]. The organism is tolerant to high pressure condition (25,000 kPa) [1]. Under anaerobic and microaerophilic conditions, *P. fumarii* is obligately chemolithoautotroph and is able to oxidize H_2_ coupled with NO_3_^-^, S_2_O_3_^2-^ and O_2_ as electron acceptors [1]. Nitrate is reduced to ammonia [1]. Organic compounds do not stimulate the growth of *P. fumarii* [1]. *P. fumarii* does not grow in media containing acetate, pyruvate, glucose, starch and elementary sulfur [1]. A highly selective enrichment method for *P. fumarii* in comparison to other members of the family *Pyrodictiaceae* is based on the use of nitrate as the sole electron acceptor [32]. Crude extracts of *P. fumarii* strain 1A^T^ cells show a strong cross-reaction with antibodies prepared against the thermosome of *Pyrodictium occultum* [32], which could suggest highly similar chaperonin protein complexes. Furthermore, a membrane-associated hydrogenase with an optimum reaction temperature of 119°C is found in cells grown on molecular hydrogen and nitrate [32]. Interestingly, succinyl-CoA reduction in *P. fumarii* is not NAD(P)H-dependent, but requires reduced methyl viologen as in *Ignicoccus hospitalis* [33,34]. In the RNA of hyperthermophiles, posttranscriptional modification has been identified as a leading mechanism of structure stabilization [35-39]. Twenty-six modified nucleosides of *P. fumarii* are detected, 11 of which are methylated in ribose [38]. *P. fumarii* exhibits a novel RNA nucleosides characterized as 1,2’-*O*-dimethylguanosine (m^1^Gm) [38].

**Table 1:** Classification and general features of *P. fumarii* 1A^T^ according to the MIGS recommendations [22] and the NamesforLife database [23].

**MIGS ID**	**Property**	**Term**	**Evidence code**
	Current classification	Domain *Archaea*	TAS [24]
		Phylum *Crenarchaeota*	TAS [25]
		Class *Thermoprotei*	TAS [26,27]
		Order *Desulfurococcales*	TAS [26,28]
		Family *Pyrodictiaceae*	TAS [29]
		Genus *Pyrolobus*	TAS [1,3]
		Species *Pyrolobus fumarii*	TAS [1,3]
		Type strain 1A	TAS [1]
	Gram stain	“negative”	TAS [1]
	Cell shape	regularly to irregularly lobed cocci, occurring singly and in short chains	TAS [1]
	Motility	none	TAS [1]
	Sporulation	none	TAS [1]
	Temperature range	90–113°C	TAS [1]
	Optimum temperature	106°C	TAS [1]
	Salinity	1%-4% (w/v) NaCl (optimum 1.7%)	TAS [1]
MIGS-22	Oxygen requirement	facultatively microaerophilic	TAS [1]
	Carbon source	CO_2_	TAS [1]
	Energy metabolism	chemolithoautotrophic	TAS [1]
MIGS-6	Habitat	abyssal deep-sea hydrothermal systems	TAS [1]
MIGS-15	Biotic relationship	free-living	NAS
MIGS-14	Pathogenicity	none	NAS
	Biosafety level	1	TAS [30]
	Isolation	rock samples from wall of a black smoker	TAS [1]
MIGS-4	Geographic location	Mid Atlantic Ridge	TAS [1]
MIGS-5	Sample collection time	1993	NAS
MIGS-4.1	Latitude	26	TAS [1]
MIGS-4.2	Longitude	- 45	TAS [1]
MIGS-4.3	Depth	3,650 m	TAS [1]
MIGS-4.4	Altitude	- 3,650 m	TAS [1]

Evidence codes - IDA: Inferred from Direct Assay (first time in publication); TAS: Traceable Author Statement (i.e., a direct report exists in the literature); NAS: Non-traceable Author Statement (i.e., not directly observed for the living, isolated sample, but based on a generally accepted property for the species, or anecdotal evidence). These evidence codes are from of the Gene Ontology project [31]. If the evidence code is IDA, the property should have been directly observed by one of the authors or an expert mentioned in the acknowledgements

### Chemotaxonomy

The S-layer of strain 1A^T^ exhibits p4 symmetry with a lattice of 18.5 nm that encloses a 40-nm-wide ‘quasi-periplasmic space’ [1]. The major core lipids of strain 1A^T^ are uncyclized glycerol-dialkyl-glycerol-tetraether (GDGT) and traces of 2,3-di-*O*-phytanyl-*sn*-glycerol (diether) [1]. Cells of strain 1A^T^ do not contain C_20_ C_25_ diethers and cyclized GDGT [1]. Non-hydrolyzed lipids contain a main spot on TLC staining blue (instead of violet) by anisaldehyde [1]. The major organic solute of strain 1A^T^ is di-*myo*-inositol phosphate (DIP) [40]. DIP and its derivatives are consistently associated with the heat stress response and therefore, are probably involved in the thermoprotection [15]. UDP-sugars are present in cells of strain 1A^T^ [40]. The structures of the two major UDP-sugars are identified as UDP-*α*-GlcNAc3NAc and UDP-*α*-GlcNAc3NAc-(4←1)-*β*-Glc*p*NAc3NAc [40]. UDP-sugars are intermediates of an *N*-linked glycosylation pathway of strain 1A^T^ [40]. Strain 1A^T^ performs a posttranscriptional modification of transfer RNA [38].

## Genome sequencing and annotation

### Genome project history

This organism was selected for sequencing on the basis of its phylogenetic position [41], and is part of the *** G****enomic* *** E****ncyclopedia of* *** B****acteria and* *** A****rchaea * project [42]. The genome project is deposited in the Genome On Line Database [14] and the complete genome sequence is deposited in GenBank. Sequencing, finishing and annotation were performed by the DOE Joint Genome Institute (JGI). A summary of the project information is shown in Table 2.

**Table 2:** Genome sequencing project information

**MIGS ID**	**Property**	**Term**
MIGS-31	Finishing quality	Finished
MIGS-28	Libraries used	Three genomic libraries: one 454 pyrosequence standard library, one 454 PE library (6 kb insert size), one Illumina library
MIGS-29	Sequencing platforms	Illumina GAii, 454 GS FLX Titanium
MIGS-31.2	Sequencing coverage	1,753.4 × Illumina; 60.4 × pyrosequence
MIGS-30	Assemblers	Newbler version 2.5, Velvet 0.7.63, phrap SPS - 4.24
MIGS-32	Gene calling method	Prodigal 1.4, GenePRIMP
	INSDC ID	CP002838
	Genbank Date of Release	*pending*
	GOLD ID	Gi02934
	NCBI project ID	48579
	Database: IMG-GEBA	2505679005
MIGS-13	Source material identifier	DSM 11204
	Project relevance	Tree of Life, GEBA

### Growth conditions and DNA isolation

*P. fumarii* 1A^T^, DSM 11204, was grown anaerobically in DSMZ medium 792 (*Pyrolobus fumarii* medium) [43] at 103°C. DNA was isolated from 0.5-1 g of cell paste using Qiagen Genomic 500 DNA Kit (Qiagen, Hilden, Germany) following the standard protocol as recommended by the manufacturer.

### Genome sequencing and assembly

The genome was sequenced using a combination of Illumina and 454 sequencing platforms. All general aspects of library construction and sequencing can be found at the JGI website [44]. Pyrosequencing reads were assembled using the Newbler assembler (Roche). The initial Newbler assembly consisting of ten contigs in one scaffold was converted into a phrap [45] assembly by making fake reads from the consensus, to collect the read pairs in the 454 paired end library. Illumina sequencing data (3,232.0 Mb) was assembled with Velvet [46] and the consensus sequences were shredded into 1.5 kb overlapped fake reads and assembled together with the 454 data. The 454 draft assembly was based on 79.2 Mb 454 draft data and all of the 454 paired end data. Newbler parameters are -consed -a 50 -l 350 -g -m -ml 20. The Phred/Phrap/Consed software package [45] was used for sequence assembly and quality assessment in the subsequent finishing process. After the shotgun stage, reads were assembled with parallel phrap (High Performance Software, LLC). Possible mis-assemblies were corrected with gapResolution [44], Dupfinisher, or sequencing cloned bridging PCR fragments with subcloning or transposon bombing (Epicentre Biotechnologies, Madison, WI) [47]. Gaps between contigs were closed by editing in Consed, by PCR and by Bubble PCR primer walks (J.-F. Chang, unpublished). A total of 12 additional reactions were necessary to close gaps and to raise the quality of the finished sequence. Illumina reads were also used to correct potential base errors and increase consensus quality using a software Polisher developed at JGI [48]. The error rate of the completed genome sequence is less than 1 in 100,000. Together, the combination of the Illumina and 454 sequencing platforms provided 1,813.8 × coverage of the genome. The final assembly contained 431,902 pyrosequence and 44,889,308 Illumina reads.

### Genome annotation

Genes were identified using Prodigal [49] as part of the Oak Ridge National Laboratory genome annotation pipeline, followed by a round of manual curation using the JGI GenePRIMP pipeline [50]. The predicted CDSs were translated and used to search the National Center for Biotechnology Information (NCBI) non-redundant database, UniProt, TIGRFam, Pfam, PRIAM, KEGG, COG, and InterPro databases. Additional gene prediction analysis and functional annotation was performed within the Integrated Microbial Genomes – Expert Review (IMG-ER) platform [51].

## Genome properties

**Figure 3:**
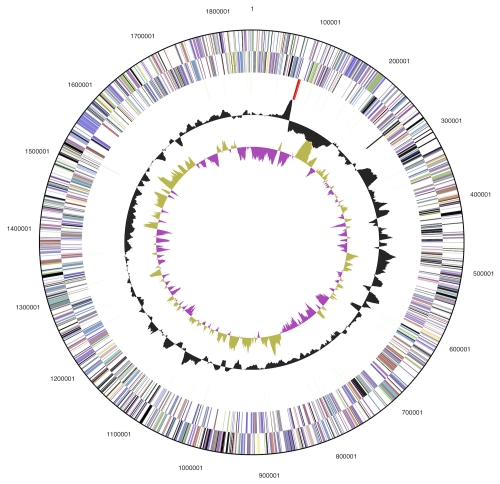
Graphical circular map of the genome. From outside to the center: Genes on forward strand (color by COG categories), Genes on reverse strand (color by COG categories), RNA genes (tRNAs green, rRNAs red, other RNAs black), GC content, GC skew.

The genome consists of a 1,843,267 bp long chromosome with a 54.9% G+C content (Table 3 and Figure 3). Of the 2,038 genes predicted, 1,986 were protein-coding genes, and 52 RNAs; 19 pseudogenes were also identified. The majority of the protein-coding genes (54.9%) were assigned a putative function while the remaining ones were annotated as hypothetical proteins. The distribution of genes into COGs functional categories is presented in Table 4.

**Table 3:** Genome Statistics

**Attribute**	**Value**	**% of Total**
Genome size (bp)	1,843,267	100.00%
DNA coding region (bp)	1,616,680	87.71%
DNA G+C content (bp)	1,012,030	54.90%
Number of replicons	1	
Extrachromosomal elements	0	
Total genes	2,038	100.00%
RNA genes	52	2.55%
rRNA operons	1	
Protein-coding genes	1,986	97.45%
Pseudo genes	19	0.93%
Genes with function prediction	1,119	54.91%
Genes in paralog clusters	82	4.02%
Genes assigned to COGs	1,325	65.01%
Genes assigned Pfam domains	1,283	62.95%
Genes with signal peptides	207	10.16%
Genes with transmembrane helices	368	18.06%
CRISPR repeats	0	

**Table 4:** Number of genes associated with the general COG functional categories

**Code**	**value**	**%age**	**Description**
J	178	12.5	Translation, ribosomal structure and biogenesis
A	2	0.1	RNA processing and modification
K	84	5.9	Transcription
L	72	5.1	Replication, recombination and repair
B	3	0.2	Chromatin structure and dynamics
D	16	1.1	Cell cycle control, cell division, chromosome partitioning
Y	0	0.0	Nuclear structure
V	10	0.7	Defense mechanisms
T	31	2.2	Signal transduction mechanisms
M	27	1.9	Cell wall/membrane/envelope biogenesis
N	10	0.7	Cell motility
Z	0	0.0	Cytoskeleton
W	0	0.0	Extracellular structures
U	19	1.3	Intracellular trafficking and secretion
O	60	4.2	Posttranslational modification, protein turnover, chaperones
C	97	6.8	Energy production and conversion
G	36	2.5	Carbohydrate transport and metabolism
E	120	8.5	Amino acid transport and metabolism
F	51	3.6	Nucleotide transport and metabolism
H	99	7.0	Coenzyme transport and metabolism
I	18	1.3	Lipid transport and metabolism
P	54	3.8	Inorganic ion transport and metabolism
Q	11	0.8	Secondary metabolites biosynthesis, transport and catabolism
R	260	18.1	General function prediction only
S	162	11.4	Function unknown
-	713	35.0	Not in COGs

## Insights from the genome sequence

Table 5 shows the whole-genome distances between *P. fumarii* and the other type strains within the order *Desulfurococcales* [15-21] as calculated using the genome-to-genome distance calculator [52-54]. As expected, the distances to the only other member of the family *Pyrodictiaceae*, *H. butylicus*, are lower than those to the members of the *Desulfurococcaceae*. This does not hold for formula 2, which is affected by saturation: if only HSPs of more strongly conserved genes are obtained, these contain, on average, a higher proportion of identical base pairs [52].

**Table 5:** Genome-to-genome distances between *P. fumarii* and the genomes of other type strains within the order*

**Reference genome**	**Formula**	**Distance**
*Aeropyrum pernix* BA000002	1	0.9809
	2	0.1414
	3	0.9836
*Desulfurococcus kamchatkensis* CP001140	1	0.9889
	2	0.1194
	3	0.9903
*Desulfurococcus mucosus* CP002363	1	0.9836
	2	0.1321
	3	0.9857
*Hyperthermus butylicus* CP000493	1	0.9514
	2	0.1632
	3	0.9593
*Ignicoccus hospitalis* CP000816	1	0.9777
	2	0.1410
	3	0.9808
*Ignisphaera aggregans* CP002098	1	0.9940
	2	0.1062
	3	0.9946
*Staphylothermus hellenicus* CP002051	1	0.9909
	2	0.1167
	3	0.9920
*Staphylothermus marinus* CP000575	1	0.9916
	2	0.1121
	3	0.9925
*Thermosphaera aggregans* CP001939	1	0.9883
	2	0.1215
	3	0.9897

*The formulas are: 1- HSP length/total length; 2- identities/HSP length; 3- identities/total length [52,53].

**Figure 4:**
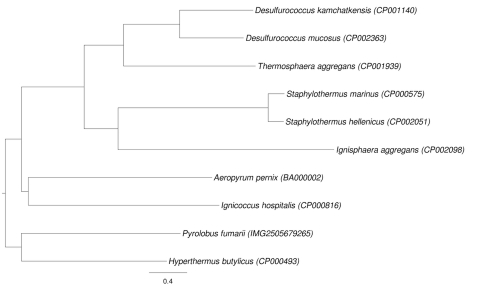
GGDC NJ tree inferred from the type strain genomes within the order *Desulfurococcales*.

Figure 4 shows a neighbor-joining tree inferred with PAUP* [13] from the logarithmized version of distance 3. The tree differs from the 16S rRNA-based tree (Figure 1) regarding the position of *Ignisphaera aggregans*, which is placed as sister group of all other *Desulfurococcaceae* by the 16S rRNA, but of *Staphylothermus* in the whole-genome tree.

**Figure 5:**
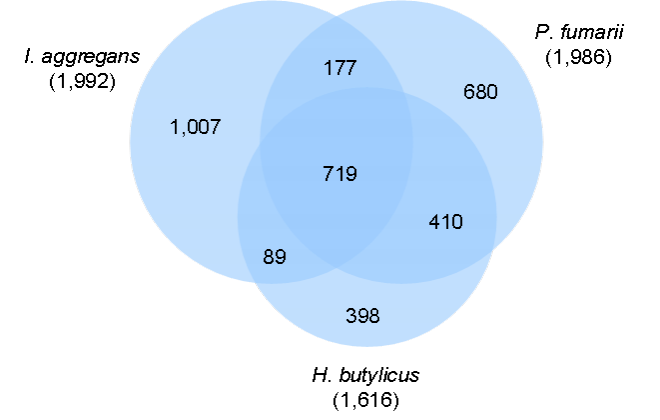
Venn diagram depicting the intersections of protein sets (total numbers in parentheses) of *P. fumarii, I. aggregans* and *H. butylicus*.

The fraction of shared genes in the genomes of *P. fumarii*, its closest neighbor *H. butylicus*, and as an outgroup *I. aggregans* (see Figure 1) is shown in a Venn diagram (Figure 5). The numbers of pairwise shared genes were calculated with the phylogenetic profiler function of the IMG-ER platform [51]. The homologous genes within the genomes were detected with a maximum E-value of 10^-5^ and a minimum identity of 30%. 719 genes (39%) are shared by *P. fumarii, I. aggregans* and *H. butylicus*. *P. fumarii* and *H. butylicus* share 410 genes, whereas *I. aggregans* shares only 89 and 177 with *H. butylicus* and *P. fumarii*, respectively, corroborating with the larger phylogenetic distance. With only 398 genes (25%) *H. butylicus* contains the smallest fraction of unique genes (and the smallest genome, 1,616 genes), while *I. aggregans* has not only the largest genome (1,992 genes), but also the highest fraction of unique genes (51%) in this set of organisms.
